# Effects of Sodium-Glucose Co-Transporter-2 Inhibition on Pulmonary Arterial Stiffness and Right Ventricular Function in Heart Failure with Reduced Ejection Fraction

**DOI:** 10.3390/medicina58081128

**Published:** 2022-08-19

**Authors:** Sencer Çamcı, Emre Yılmaz

**Affiliations:** Department of Cardiology, Faculty of Medicine, Giresun University, 28100 Giresun, Turkey

**Keywords:** heart failure, pulmonary arterial stiffness, right ventricular function, SGLT2 inhibitors

## Abstract

*Background and Objectives:* In addition to left ventricular (LV) functions, right ventricular (RV) functions and pulmonary arterial stiffness (PAS) may be adversely affected in patients with heart failure with reduced ejection fraction (HFrEF). Sodium-glucose co-transporter-2 (SGLT2) inhibitor therapy positively affects LV functions as well as having functional and symptomatic benefits in HFrEF patients. In this study, we aimed to evaluate the effects of SGLT2 inhibitor treatment on RV function and PAS in HFrEF patients. *Materials and* *Methods:* 168 HFrEF patients with New York Heart Association (NYHA) class ≥2 symptoms despite optimal medical treatment and who were started on SGLT2 inhibitor therapy were included in this retrospective study. NYHA classification, N-terminal pro-B-type natriuretic peptide (NT-proBNP) levels, Minnesota Living with Heart Failure Questionnaire (MLWHFQ) scores, laboratory tests, and transthoracic echocardiography (TTE) measurements were recorded before treatment and at the end of the 6-month follow-up. *Results:* The mean age of the patients was 62.7 ± 11.4 years, and 38 (22.6%) were women. RV function (RV fractional area change (FAC) (33.8 ± 6.4% vs. 39.2 ± 7.3%, *p* < 0.001); tricuspid annular plane systolic excursion (TAPSE) (18.4 ± 3.8 mm vs. 19.6 ± 3.6 mm, *p* < 0.001); RV S’ (10 (8 − 13) cm/s vs. 13 (10 − 16) cm/s, *p* < 0.001); RV myocardial performance index (RV MPI) (0.68 ± 0.12 vs. 0.59 ± 0.11, *p* < 0.001); mean pulmonary artery pressure (mPAP) (39.6 ± 7.8 mmHg vs. 32 ± 6.8 mmHg, *p* = 0.003)) and PAS (24.2 ± 4.6 kHz/ms vs. 18.6 ± 3.1 kHz/ms, *p* < 0.001) values at the 6-month follow-up after SGLT2 inhibitor therapy significantly improved. It was found that SGLT2 inhibitor treatment provided significant improvement in NYHA classification, MLWHFQ scores, and NT-proBNP levels (2876 ± 401 vs. 1034 ± 361, *p* < 0.001), and these functional and symptomatic positive changes in HFrEF patients were significantly correlated with positive changes in LVEF, PAS, and RV functional status. *Conclusions:* SGLT2 inhibitor treatment results in symptomatic and functional well-being in HFrEF patients, as well as positive changes in RV function and PAS.

## 1. Introduction

Heart failure (HF) is a chronic inflammatory syndrome with typical symptoms and signs, which reduces the quality of life and increases the risk of mortality and morbidity [1]. In patients with heart failure with reduced ejection fraction (HFrEF), right ventricular (RV) dysfunction is frequently observed in addition to left ventricular (LV) dysfunction. RV dysfunction can be considered a predictor of progression and poor prognostic outcomes in HF patients [2]. Similarly, an improvement in RV function was associated with more favorable outcomes in HF patients [3].

Pulmonary arterial stiffness (PAS) is an index that is higher in HFrEF patients than in the healthy population and allows the evaluation of the structural and functional status of the pulmonary vascular bed [4,5]. DAPA-HF and EMPEROR-Reduced studies have shown that sodium-glucose co-transporter-2 (SGLT2) inhibitor treatment improved the quality of life and alleviated heart failure symptoms in HFrEF patients who described symptoms in New York Heart Association (NYHA) class II-IV despite optimal medical therapy [6,7].

SGLT2 inhibitor therapy has been shown to increase LV ejection fraction and contractile reserve and improve LV adverse remodeling [8]. However, data on the effects of treatment on RV function and pulmonary vascular bed in HFrEF patients followed up with SGLT2 inhibitor therapy are limited. In this study, we aimed to evaluate the effects of SGLT2 inhibitor treatment on RV function and PAS in HFrEF patients.

## 2. Materials and Methods

### 2.1. Patients

HFrEF patients with NYHA class ≥2 symptoms followed up from our cardiology clinic between January 2020 and April 2022 were evaluated for our single-center retrospective study. Inclusion criteria were: >18 years of age, LVEF ≤ 40%, and presence of NYHA class 2–4 symptoms despite optimal medical treatment. Exclusion criteria were determined as follows (in the last 6 months before the initiation of therapy and during the 6-month follow-up period): (i) implantation of a cardiac pacemaker, implantable cardioverter-defibrillator (ICD), or cardiac resynchronization therapy (CRT); (ii) coronary artery bypass graft surgery; (iii) surgical or percutaneous interventions for valve disease; (iv) percutaneous coronary intervention for acute coronary syndrome; (v) inflammatory, autoimmune and infectious diseases under follow-up and treatment; (vi) ongoing hematological diseases and malignancies; and (vii) advanced stage liver and kidney failure (glomerular filtration rate < 30 mL/min/1.73 m^2^). After applying the inclusion and exclusion criteria, a total of 168 HFrEF patients were included in our study. This study followed the principles stated in the Declaration of Helsinki and was approved by the local ethics committee (Ordu University Clinical Research Ethics Committee No: 2022/125). Written informed consent was taken from all participants.

### 2.2. Study Protocol

Baseline demographic data, baseline laboratory tests performed before the decision for SGLT2 treatment, vital signs, N-terminal pro-B-type natriuretic peptide (NT-proBNP), New York Heart Association (NYHA) classification, Minnesota Living with Heart Failure Questionnaire (MLWHFQ) scores, body mass index (BMI), electrocardiographic (ECG) and transthoracic echocardiographic (TTE) measurements of the patients included in our study were recorded. Patients who were given SGLT2 inhibitor therapy were invited to follow-up visits at the first, third, and sixth months to monitor for treatment compliance. Optimal medical treatment was restored according to the volume load, functional capacity and symptoms of the patients at the control visits. Diuretic doses were downregulated in patients with reduced volume load. Relevant measurements were repeated at each visit. In the analyses, baseline measurements before SGLT2 inhibitor treatment and at the six-month follow-up visit measurements were compared. Patients who did not attend or could not be reached at the control visits were excluded from the study. Tolerability of SGLT2 inhibitor therapy or its regular use for at least 6 months was considered to be one of the important conditions for inclusion in the study.

### 2.3. Definitions

In line with the 2021 European Society of Cardiology (ESC) heart failure guidelines recommendations, optimal medical therapy (OMT) was defined as angiotensin converting enzyme (ACE) inhibitors/angiotensin receptor blockers (ARB)/sacubitril valsartan (SV) + beta-blocker + mineralocorticoid receptor antagonist (MRA) and SGLT2 inhibitors. The fact that the patients were symptomatic despite OMT was expressed after the maximum dose of the OMT drug group they could tolerate [1]. BMI measurements were calculated with the formula weight (kg)/(height (m))^2^. The Minnesota Living with Heart Failure Questionnaire (MLWHFQ) contains 21 questions that assess the effects of heart failure symptoms and signs on social relationships, physical and sexual activity, work, and emotions. The answer to each question was chosen from a scale of 0 (not at all) to 5 (very much); the higher the score, the worse or more restricted the quality of life [9].

Hypertension (HT) was defined as ongoing medication use with a previous diagnosis of HT, or systolic blood pressure (SBP) ≥ 140 mmHg or diastolic blood pressure (DBP) ≥ 90 mmHg (the mean of two consecutive measurements). Diabetes mellitus (DM) was defined as the use of anti-diabetic drugs or a fasting blood glucose level of ≥126 mg/dL or HbA1c ≥ 6.5% on at least two measurements. Hyperlipidemia was defined as low-density lipoprotein (LDL) cholesterol levels ≥130 mg/dL or ongoing statin use.

### 2.4. Echocardiography Measurements

All echocardiographic examinations were performed by experienced operators (Vivid S5, GE, Horten, Norway). Echocardiographic examinations were performed in the left lateral decubitus position after resting for at least 15 min. All measurements were taken in three consecutive cycles and mean values were calculated. Parasternal long and short axis views and apical views were used as standard viewing windows. All echocardiographic evaluations were performed according to the recommendations of the American Society of Echocardiography/European Association of Cardiovascular Imaging guidelines [10].

Left ventricular end-diastolic and end-systolic diameters (LVEDd, LVESd) were measured from the parasternal long-axis view using M-mode. Left ventricular end-diastole and end-systole volumes were measured from the apical four-chamber and two-chamber views. Ejection fraction measurements were made using the Modified Simpson method. Left ventricular outflow tract (LVOT) diameter was measured in the parasternal long-axis view in systole. The left ventricular outflow tract velocity time integral (LVOT-VTI) was calculated using pulsed-wave (PW) Doppler on the LVOT. The cardiac index (L/min/m^2^) was calculated with the formula (LVOT − VTI × LVOT area) × heart rate (beats/min)/BMI (kg/m^2^). RV end-diastolic diameters were calculated from the middle and basal segments in an apical four-chamber view. RV end-diastolic (RVEDa) and end-systolic areas (RVESa) were calculated from the apical four-chamber view. RV fractional area change (RV FAC) was calculated with the formula (RVEDa − RVESa)/RVEDa. Tricuspid annular plane systolic excursion (TAPSE) was measured as the displacement of the tricuspid lateral annulus between systole and diastole in M-mode. RV peak systolic S’ velocity was calculated by tissue Doppler velocity of the lateral tricuspid annulus. Mean pulmonary artery pressure (mPAP) was calculated from systolic PAP using the following formula: mPAP = 0.61 × sPAP + 2 mmHg [11]. RV myocardial performance index (RV MPI) was calculated with the formula (isovolumic contraction time (IVCT) + isovolumic relaxation time (IVRT))/ejection time (ET). Components of the RV MPI formula were evaluated with PW tissue Doppler imaging (TDI).

**Figure 1:**
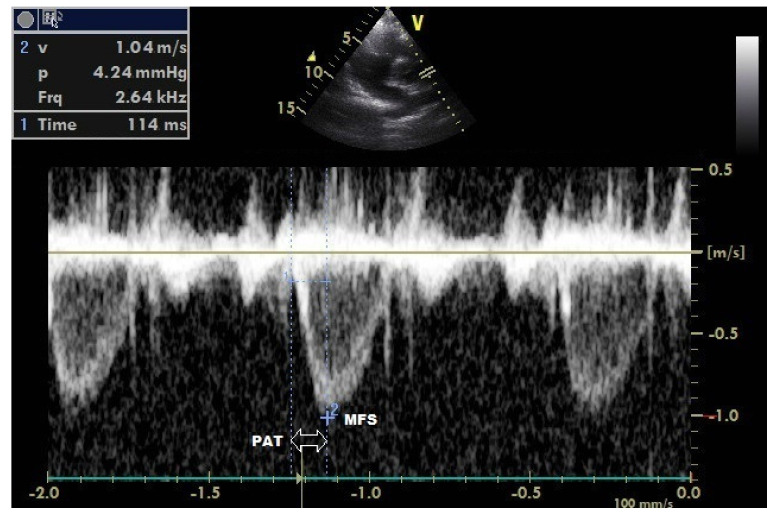
Evaluation of pulmonary arterial stiffness (PAS) by echocardiography. PAS (kHz/ms) = maximum frequency shift (MFS)/pulmonary flow acceleration time (PAT).

In parasternal short axis imaging, pulsed Doppler recordings of pulmonary artery blood flow were taken by placing a sample volume 1 cm below the pulmonary valve annulus. Peak velocity (maximum frequency shift = MFS) of pulmonary flow was calculated by averaging at least three consecutive beat measurements. Again, the time from the onset of pulmonary flow ejection to the peak (pulmonary flow acceleration time = PAT) was calculated by averaging at least three consecutive pulse measurements (Figure 1). PAS (kHz/ms) was calculated with the formula = maximum frequency shift (MFS)/pulmonary flow acceleration time (PAT) [4].

TTE measurements made before SGLT2 inhibitor treatment and at the 6-month follow-up visit were compared in the analyses.

### 2.5. Statistical Analysis

Statistical analyses were performed using SPSS 21 for Windows (SPSS Inc., Chicago, IL, USA). The continuous variables were tested for a normal distribution using the Kolmogorov-Smirnov test. Non-normally distributed data were presented as the median with an interquartile range and compared with Wilcoxon’s signed-rank test. Normally distributed data were expressed as mean ± standard deviation and compared with the Student’s *t*-test, categorical variables were calculated as percentages and compared with χ^2^ test. Linear correlations were determined by measuring Pearson’s correlation coefficient. A *p* < 0.05 was considered statistically significant

## 3. Results

The mean age of the 168 HFrEF patients included in our study was 62.7 ± 11.4, and 38 (22.6%) were women. In the study population, 58.3% of the etiology of heart failure was ischemic-based, 63.7% had HT, 32.1% had DM, 67.8% had a history of coronary artery disease, and 50% had dyslipidemia. It was observed that 92.8% of our study patients used β-blocker, 51.7% ACE inhibitor, 42.2% SV, and 63.1% spironolactone before SGLT2 inhibitor treatment. Baseline demographic and clinical data of the study patients are presented in Table 1.

The comparison of baseline and 6-month control data before SGLT2 inhibitor therapy is presented in Table 1. There was no significant change in the BMI, weight, vital signs, and laboratory tests of the patients after 6 months of follow-up. There was a significant increase in albumin level compared with pretreatment (baseline: 4.1 ± 0.5 g/dL; 6-month follow-up: 4.9 ± 0.65 g/dL (*p* = 0.022)). While the mean NT-proBNP level was 2876 ± 401 pg/mL before treatment, it was observed to decrease to 1034 ± 361 pg/mL levels after treatment (*p* < 0.001). While the rate of patients with symptoms in NYHA class III and IV before treatment was calculated as 58.9%, this rate decreased to 21.4% in the sixth month of the controls after treatment (*p* < 0.001). It was determined that the MLWHFQ score decreased significantly in the 6-month control period compared with pre-treatment (baseline: 32.4 ± 6.2; 6-month follow-up: 24.6 ± 4.4 (*p* < 0.001)).

**Figure 2:**
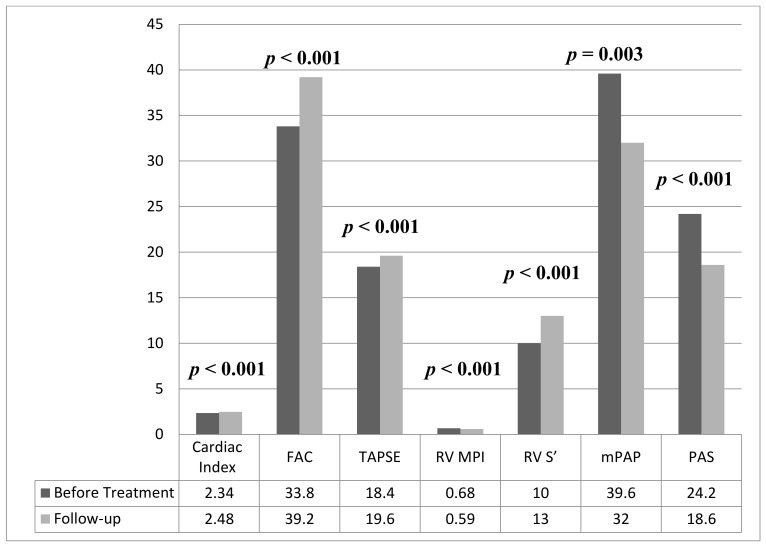
Changes in right ventricular functional assessment and pulmonary arterial stiffness measurements before and after SGLT2 inhibitor treatment at the 6-month control period. FAC fractional area change, TAPSE tricuspid annular plane systolic excursion, RV MPI right ventricle myocardial performance index, mPAP mean pulmonary artery pressure, and PAS pulmonary arterial stiffness.

Baseline and 6-month follow-up TTE data are presented in Table 2. LVEF and cardiac index were found to increase significantly after SGLT2 inhibitor treatment (LVEF baseline: 27.5 ± 4.7%; follow-up: 29.2 ± 4.2% (*p* < 0.001), cardiac index baseline: 2.34 ± 0.46 L/min/m^2^; and follow-up: 2.48 ± 0.42 L/min/m^2^ (*p* < 0.001)). It was observed that LVEDd, LVESd, left ventricular end-diastolic and end-systolic volumes (LVEDV, LVESV) decreased significantly at the 6-month follow-up compared with pretreatment (*p* = 0.002; 0.008; <0.001; 0.001, respectively). The parameters we used in the functional evaluation of the right ventricle at the 6-month follow-up were compared to baseline and it was found that: (i) RV FAC (33.8 ± 6.4% vs. 39.2 ± 7.3%, *p* < 0.001), TAPSE (18.4 ± 3.8 mm vs. 19.6 ± 3.6 mm, *p* < 0.001) and RV S’ (10 (8 − 13) cm/s vs. 13 (10 − 16) cm/s, *p* < 0.001) significantly increased; (ii) RV MPI (0.68 ± 0.12 vs. 0.59 ± 0.11, *p* < 0.001), mPAP (39.6 ± 7.8 mmHg vs. 32 ± 6.8 mmHg, *p* = 0.003) and PAS (24.2 ± 4.6 kHz/ms vs. 18.6 ± 3.1 kHz/ms, *p* < 0.001) significantly decreased (Figure 2).

Table 3 shows the correlation results of MLWHFQ score, NYHA classification, and NT-proBNP levels before and after treatment at 6 months with the changes in TTE parameters, which are the criteria we used to evaluate the functional, symptomatic and prognostic course of HFrEF patients. LVEF, cardiac index, RV FAC, TAPSE, and RV S’ were found to be negatively correlated with all three criteria. RV MPI and PAS measurement changes were found to be significantly positively correlated with MLWHFQ, NYHA, and NT-proBNP changes.

## 4. Discussion

To the best of our knowledge, this is the first study to evaluate the effect of SGLT2 therapy on PAS and RV function in patients with HFrEF. A decrease in PAS values and improvement in RV function were detected at the end of the 6-month follow-up in patients who received SGLT2 treatment. When the pre-treatment and post-treatment 6-month control changes were compared; MLWHFQ score, NYHA classification, and NT-proBNP levels, which are the criteria we used to evaluate the functional, symptomatic and prognostic course of HFrEF patients, were found to be negatively correlated with LVEF, cardiac index, RV FAC, TAPSE and RV S’, and positively correlated with RV MPI and PAS values.

Impaired LV functions also negatively affect RV function. The increase in pressure due to LV dysfunction is reflected in the pulmonary vascular system, and indirectly the increase in afterload worsens RV function [12]. Worsening of RV function indicates progression in HF and is associated with a poor prognosis in patients with HFrEF [2]. Echocardiography studies on the evaluation of RV functioning have been increasing in recent years [13]. Improvements in RV function are indicative of an improvement in LV functions and a good prognostic course in patients with HFrEF [3]. The main parameters used to evaluate RV function in the literature are RV FAC, TAPSE, RV S’, and RV MPI [10].

PAS is an index developed to evaluate the structural features and functions of the pulmonary vascular bed and indicates pulmonary artery elasticity [4]. Clinical conditions triggering remodeling of the pulmonary vascular structure may cause an increase in PAS. It has been shown that PAS is higher in HFrEF compared with the normal population [4,5,14]. Reactive changes occur in pulmonary vascular structures accompanied by intimal fibrosis, smooth muscle cell proliferation, and elastic fiber abnormalities as a result of chronic elevations of LV filling pressure in HFrEF patients. Chronic inflammation and hypoxia, endothelial dysfunction, an increase in endothelin-1 expression, a decrease in nitric oxide levels and upregulation of neurohormones can be shown as the causes of these changes. The remodeling process resulting from this complex interaction between cells and extracellular matrix components can cause stiffening of the vascular bed and a progressive decrease in pulmonary artery compliance, resulting in elevated PAS values [15,16,17,18,19]. As a result of increasing PAS, an increase in RV afterload occurs and RV function deteriorate over time [20]. In a study conducted on HFrEF patients, PAS values were found to be higher than in the control group. This supports the idea that the pulmonary vascular bed is affected in HFrEF patients [5]. Therefore, the evaluation of PAS is important in the management of HFrEF patients. The gold standard measurement method is right heart catheterization, but the fact that it is an invasive method makes its routine use difficult. For this reason, noninvasive measurement methods, such as computed tomography (CT), magnetic resonance (MR), and echocardiography have been developed [21,22]. There are study designs that have achieved successful results by comparing noninvasive diagnostic methods, especially cardiac MR, with right heart catheterization for assessment of RV function and PAS. For example, Kang et al. [22] reported that the pulmonary artery flexibility index calculated by MR in 35 pulmonary arterial hypertension patients was correlated with the pulmonary arterial stiffness calculated by right heart catheterization. Tong et al. [23] compared cardiac MR, speckle tracking echo, and classical 2D echo measurements (TAPSE, FAC, and S’) evaluating right ventricular function in 29 patients with dilated cardiomyopathy. In this study, cardiac MR tissue tracking (CMR-TT) and speckle tracking echocardiography (STE) global longitudinal strain (GLS) was found to be superior to other measurement parameters. However, in the subgroup analysis of patients with right ventricular systolic dysfunction, the diagnostic statistical data obtained for FAC, TAPSE and S’ from the diagnosis of patients with RVEF <45% were non-inferior to cardiac MR and strain echo data. Echocardiography, which is a more accessible imaging method in daily practice compared to CT and MR, does not require the use of radiation or contrast material, is completely non-invasive, and has proven its success in PAS measurement. In a study conducted in patients with pulmonary hypertension caused by congenital heart disease, PAS values measured by right heart catheterization were found to be compatible with values measured by echocardiography [4]. Afterward, PAS measurements were used again in many clinical studies and proved its success [5,24,25,26]. We also used echocardiography for PAS measurement because it was more advantageous in our study.

Many studies have shown that SGLT2 inhibitors reduce the risk of cardiovascular death and improve cardiovascular outcomes in type 2 DM patients [27,28]. In these studies, the cardiac ameliorating effect of SGLT2 and the reduction of adverse cardiovascular events occur much more rapidly than the glycemic control effect. This suggested that mechanisms other than hyperglycemia control were responsible for its positive cardiac effect. In the post-hoc analyses of clinical studies, it was shown that the improvements in HBA1c were not related to the cardiac positive effects of SGLT2 [27,28,29,30]. As evidence for this, studies in HFrEF patients showed a lower risk of worsening heart failure or death from cardiovascular causes in patients receiving SGLT2, regardless of the presence or absence of diabetes, compared with those receiving the placebo [6,7]. Our observations strongly support the role of SGLT2 inhibitors in the treatment of patients with HFrEF, regardless of their glycemic status. The administration of empagliflozin to nondiabetic HF patients significantly improves LV volumes, LV mass, LV systolic function, functional capacity, and quality of life compared with the placebo. This improvement in LV function in empagliflozin-treated patients is supported by the reduction in plasma levels of NT-proBNP [31]. Consistent with the results obtained by the authors, we also found a significant increase in LVEF and cardiac index, and a significant decrease in LV end-diastolic and systolic diameters and volumes in our HFrEF patients treated with SGLT2 inhibitor as a result of the 6-month follow-up. SGLT2 inhibitors induce a shift in myocardial metabolism from glucose utilization to the consumption of fatty acids, ketone bodies, and branched-chain amino acids that increase myocardial energetics. Improving myocardial energetics increases LV systolic function and improves reverse LV remodeling [8]. SGLT2 inhibitors also have significant diuretic and natriuretic effects. Thus, LV preload decreases and LV volumes recover over time [31]. By reducing arterial stiffness, they also reduce afterload and increase cardiac efficiency [32]. Many other mechanisms have been identified that may be responsible for the cardiac beneficial effects of SGLT2 inhibitors. SGLT2 inhibitors provide blood pressure reduction, help weight loss, suppress the sympathetic nervous system, reduce epicardial fat thickness, reduce hyperuricemia, and inhibit cardiac Na^+^/H^+^ exchanger [33,34,35,36].

SGLT2 inhibitors have direct effects on vascular cells. They positively affect the proliferation, migration, differentiation, and senescence of endothelial cells. They have powerful antioxidant and anti-inflammatory effects. They improve endothelium-dependent vasodilation by increasing the bioavailability of nitric oxide produced from the endothelium. They inhibit the molecular changes that cause early atherogenesis. They inhibit the contraction, proliferation and migration of vascular smooth muscle cells. As a result, vascular remodeling decreases arterial wall stiffness and vascular resistance decreases [37,38,39,40,41]. The positive PAS change we obtained in HFrEF patients with SGLT2 inhibitor treatment supports the positive effects of SGLT2 inhibitors on arterial stiffness.

Previously, there have been studies on the effect of SV, a new generation heart failure drug, on RV function and PAS. In the study of Landolfo et al. [42], an improvement was found in sPAP values after 12 months of SV treatment in HFrEF patients. In the study by Correale et al. [43], 6 months of treatment with SV was associated with an improved RV function in HFrEF. Improvement in RV function was estimated by evaluating baseline RV S’ and right atrial volume. In the study of Yenerçağ et al. [26], an improvement was found in PAS and RV function after 6 months of SV treatment.

In our study, a significant decrease was observed in the NYHA class, which is an indicator of HF symptomatic status and functional capacity, in the MLWHFQ score, which is an indicator of HF quality of life, and in the levels of NT-proBNP, which is the biochemical parameter of HF severity. There was an increase in RV FAC, TAPSE, and RV S’ values used in the evaluation of right ventricular functions, and a decrease in RV MPI values, and it was determined that right ventricular echocardiographic improvements correlated with clinical and biochemical improvement. Again, it was found that PAS values, which were strongly associated with RV function [14], decreased and this decrease was positively correlated with the decrease in NYHA classification, MLWHFQ score, and NT-proBNP levels. These results suggest that SGLT2 inhibitors have ameliorative effects on the pulmonary vascular system and RV function in HFrEF patients.

### Limitations

There are some limitations of our study. In our study, no randomization was performed that compared the SGLT2 inhibitor and the placebo. It was conducted in a relatively small cohort and had a single-center retrospective design. Another important limitation was the lack of right heart catheterization or cardiac magnetic resonance imaging to evaluate pulmonary arterial stiffness, pulmonary artery pressure, and right ventricular function. In addition, patients who did not attend or could not be reached at control visits were excluded from the study. This might have influenced our results.

## 5. Conclusions

As a result of the 6-month follow-up of HFrEF patients receiving SGLT2 inhibitor therapy, PAS, which is the evaluation criteria of the pulmonary vascular bed, and RV FAC, TAPSE, RV MPI, and RV S’, which are the RV functional evaluation criteria, showed a significant positive change. Positive changes in RV function and PAS are also significantly correlated with positive functional and symptomatic outcomes in HFrEF patients. SGLT2 inhibitor therapy not only results in symptomatic and functional well-being in HFrEF patients but also provides positive changes in RV function and pulmonary arterial stiffness, directly or indirectly.

## Figures and Tables

**Table 1:** Baseline demographic and clinical parameters of the study population. Laboratory and clinical parameters before and after SGLT2 inhibitor treatment.

Parameters	Baseline (*n* = 168)		
Age (years)	62.7 ± 11.4		
Gender: female, *n* (%)	38 (22.6%)		
Ischemic heart failure, *n* (%)	98 (58.3%)		
Hypertension, *n* (%)	107 (63.7%)		
Diabetes mellitus, *n* (%)	54 (32.1%)		
Coronary artery disease, *n* (%)	114 (67.8%)		
Dyslipidemia *n* (%)	84 (50%)		
COPD *n* (%)	38 (22.6%)		
Atrial fibrillation	37 (22%)		
β-Blocker use, *n* (%)	156 (92.8%)		
ACE inhibitor use, *n* (%)	87 (51.7%)		
Sacubitril/valsartan use, *n* (%)	71 (42.2%)		
Spironolactone use, *n* (%)	106 (63.1%)		
Statin use, *n* (%)	37 (22%)		
Ivabradine use, *n* (%)	59 (35.1%)		
Diuretic use, *n* (%)	156 (92.8%)		
Digoxin use, *n* (%)	28 (16.6%)		
	**Baseline**	**Follow-Up**	***p*** **-Value**
BMI (kg/m^2^)	26.9 ± 4.2	26.7 ± 4.7	0.524
Weight (kg)	74.6 ± 13.	75.1 ± 12.	0.654
SBP (mmHg)	125.4 ± 22	124.8 ± 23	0.257
DBP (mmHg)	68.2 ± 11	66.9 ± 11	0.328
Sodium (mmol/L)	138.6 ± 4.56	138.5 ± 4.34	0.265
Potassium (mmol/L)	4.4 (3.2–5.8)	4.5 (3.3–5.8)	0.446
Hematocrit (%)	38.9 ± 4.5	39.1 ± 4.7	0.266
eGFR (mL/min/1.73 m^2)^	66.9 ± 14.4	67.6 ± 14.6	0.289
Total cholesterol (mg/dL)	162.16 ± 11.56	164.32 ± 12.16	0.346
LDL (mg/dL)	108 (52–140)	109 (56–148)	0.418
CRP (mg/L)	4.8 (2.5–12.1)	4.9 (2.9–10.9)	0.186
HbA1c (%)	6.9 ± 1.5	6.7 ± 1.5	0.322
Albumin (g/dL)	4.1 ± 0.5	4.9 ± 0.6	0.022
NT-proBNP (pg/mL)	2876 ± 401	1034 ± 361	<0.001
NYHA class			<0.001
Class I, *n* (%)	0	39 (23.2%)	
Class II, *n* (%)	69 (41.1%)	93 (55.4%)	
Class III, *n* (%)	87 (51.8%)	36 (21.4%)	
Class IV, *n* (%)	12 (7.1%)	0	
MLWHFQ score	32.4 ± 6.2	24.6 ± 4.4	<0.001
Heart rate (bpm)	74.1 ± 4.8	73.8 ± 4.4	0.412
QRS (ms)	122.4 ± 8.9	124.1 ± 9.2	0.162

COPD chronic obstructive pulmonary disease, ACE angiotensin-converting enzyme, BMI body mass index, SBP systolic blood pressure, DBP diastolic blood pressure, eGFR estimated glomerular filtration rate, LDL low-density lipoprotein, CRP C reactive protein, NT-proBNP N-terminal pro-B-type natriuretic peptide, NYHA New York Heart Association, and MLWHFQ Minnesota Living with Heart Failure Questionnaire.

**Table 2:** Echocardiographic parameters before and after SGLT2 inhibitor treatment.

Parameters	Baseline	Follow-Up	*p*-Value
LVEF (%)	27.5 ± 4.7	29.2 ± 4.2	<0.001
LVEDd (mm)	61 (58–63)	58 (56–61)	0.002
LVESd (mm)	48 ± 8.4	46 ± 8.6	0.008
LVEDV (mL)	157.5 ± 31.2	147.4 ± 29.8	<0.001
LVESV (mL)	104.2 ± 24.8	94.8 ±23.1	0.001
Mitral regurgitation, ≥Grade II, (%)	72 (42.8)	68 (40.4)	0.128
Tricuspid regurgitation, ≥Grade II, (%)	129 (76.7)	122 (72.6)	0.283
Cardiac index (L/min/m^2^)	2.34 ± 0.46	2.48 ± 0.42	<0.001
RV mid-diameter (mm)	33 (31–36)	34 (31–37)	0.089
RV basal-diameter (mm)	30 (28–33)	29 (26–31)	0.009
RV FAC, (%)	33.8 ± 6.4	39.2 ± 7.3	<0.001
TAPSE (mm)	18.4 ± 3.8	19.6 ± 3.6	<0.001
RV MPI	0.68 ± 0.12	0.59 ± 0.11	<0.001
RV S’ (cm/s)	10 (8–13)	13 (10–16)	<0.001
mPAP (mmHg)	39.6 ± 7.8	32 ± 6.8	0.003
PAS (kHz/ms)	24.2 ± 4.6	18.6 ± 3.1	<0.001

LVEF left ventricular ejection fraction, LVEDd left ventricular end-diastolic diameter, LVESd left ventricular end-systolic diameter, LVEDV left ventricular end-diastolic volume, LVESV left ventricular end-systolic volume, RV right ventricle, RV FAC right ventricular fractional area change, TAPSE tricuspid annular plane systolic excursion, RV MPI right ventricle myocardial performance index, mPAP mean pulmonary artery pressure, PAS pulmonary arterial stiffness.

**Table 3:** Correlation relationship between changes in TTE measurement parameters and changes in MLWHFQ score, NYHA classification, and NT-proBNP levels as a result of the 6-month follow-up.

	MLWHFQ Score		NYHA Class		NT-proBNP	
	r	*p*-Value	r	*p*-Value	r	*p*-Value
LVEF	−0.268	0.002	−0.284	0.001	−0.306	<0.001
LVEDd	0.122	0.238	0.101	0.252	0.224	0.024
LVESd	0.186	0.306	0.221	0.032	0.252	0.011
LVEDV	0.248	0.009	0.206	0.398	0.236	0.346
LVESV	0.212	0.502	0.258	0.009	0.269	0.007
Cardiac index	−0.356	<0.001	−0.362	<0.001	−0.388	<0.001
RV mid-diameter	−0.101	0.682	−0.096	0.861	−0.142	0.509
RV basal-diameter	0.208	0.526	0.169	0.564	0.194	0.428
RV FAC	−0.398	<0.001	−0.402	<0.001	−0.392	<0.001
TAPSE	−0.424	<0.001	−0.418	<0.001	−0.486	<0.001
RV MPI	0.468	<0.001	0.456	<0.001	0.480	<0.001
RV S’	−0.398	<0.001	−0.427	<0.001	−0.419	<0.001
mPAP	0.242	0.082	0206	0.199	0.292	0.001
PAS	0.496	<0.001	0.484	<0.001	0.492	<0.001

MLWHFQ Minnesota Living with Heart Failure Questionnaire, NYHA New York Heart Association, NT-proBNP N-terminal pro-B-type natriuretic peptide, LVEF left ventricular ejection fraction, LVEDd left ventricular end-diastolic diameter, LVESd left ventricular end-systolic diameter, LVEDV left ventricular end-diastolic volume, LVESV left ventricular end-systolic volume, RV right ventricle, RV FAC right ventricular fractional area change, TAPSE tricuspid annular plane systolic excursion, RV MPI right ventricle myocardial performance index, and mPAP: mean pulmonary artery pressure, PAS pulmonary arterial stiffness.

## Data Availability

Data are available on request due to privacy.
